# Genome Sequence of African Swine Fever Virus BA71, the Virulent Parental Strain of the Nonpathogenic and Tissue-Culture Adapted BA71V

**DOI:** 10.1371/journal.pone.0142889

**Published:** 2015-11-30

**Authors:** Javier M. Rodríguez, Leticia Tais Moreno, Alí Alejo, Anna Lacasta, Fernando Rodríguez, María L. Salas

**Affiliations:** 1 Centro de Biología Molecular Severo Ochoa (Consejo Superior de Investigaciones Científicas-Universidad Autónoma de Madrid), Universidad Autónoma de Madrid, Madrid, Spain; 2 Centro Nacional de Microbiología, Instituto Nacional de Salud Carlos III, Majadahonda, Madrid, Spain; 3 Parque Científico de Madrid, Campus de Cantoblanco, Madrid, Spain; 4 Centre de Recerca en Sanitat Animal (CReSA)—Institut de Recerca i Tecnologia Agroalimentàries (IRTA), Bellaterra, Barcelona, Spain; University of Minnesota, UNITED STATES

## Abstract

The strain BA71V has played a key role in African swine fever virus (ASFV) research. It was the first genome sequenced, and remains the only genome completely determined. A large part of the studies on the function of ASFV genes, viral transcription, replication, DNA repair and morphogenesis, has been performed using this model. This avirulent strain was obtained by adaptation to grow in Vero cells of the highly virulent BA71 strain. We report here the analysis of the genome sequence of BA71 in comparison with that of BA71V. They possess the smallest genomes for a virulent or an attenuated ASFV, and are essentially identical except for a relatively small number of changes. We discuss the possible contribution of these changes to virulence. Analysis of the BA71 sequence allowed us to identify new similarities among ASFV proteins, and with database proteins including two ASFV proteins that could function as a two-component signaling network.

## Introduction

African swine fever (ASF) is a hemorrhagic and frequently lethal disease of domestic pigs caused by African swine fever virus (ASFV), a complex enveloped deoxyvirus of icosahedral morphology, whose genome is a lineal double-stranded DNA molecule with covalently closed ends and terminal inverted repeats (TIR), identical at both ends. The remainder of the genome corresponds to a unique sequence, interrupted in some cases by short regions of tandem repeats [1,2]. The size of the genome varies between different isolates, oscillating between 170 and 190 kbp. Three main regions with respect to the frequency and nature of the changes observed have been identified. A central region of about 125 kbp, which shows differences in size that are less than 1.5% of the total size, and two highly variable regions at the ends [3,4]. Inside the central constant region there are zones of high localized variability, produced by the compression and expansion of stretches of tandem repeats, some of which are used for the enhanced discrimination between isolates [5]. However, most of the genetic variation is due to changes in the number and sequence of members of the ASFV multigene families (MGF), located at both ends of the genome, where they delimit the left and right variable regions (LVR and RVR, respectively) [6]. The ASFV strain BA71V encodes 32 genes corresponding to six different families. Five of the multigene families are designated according to the approximate size in amino acids of the proteins belonging to the family. In this isolate, MGF 110 (5 genes) and MGF 300 (4 genes) are localized exclusively at the LVR, while MGF 100 (2 genes) is present at the RVR and MGF 360 (12 genes) and 505 (8 genes) are found at both ends. Only one gene of the MGF p22, located in the 5’-end of the genome, is present in this strain.

The course of the infection in domestic pigs depends on the ASFV isolate. Highly virulent isolates cause a fulminating disease with 100% mortality in a few days, while attenuated strains may produce a mild disease or subclinical chronic infections [7,8]. It has been suggested that members of multigene families 360 and 505 are determinants of the virulence and host range of ASFV. By means of marker rescue experiments and the construction of deletion mutant virus, it has been shown that the deletion of two genes of family 505 and eight genes of family 360 completely abrogates the capacity of a virulent virus to infect macrophages [9]. Furthermore, six of these genes appear to be also involved in the maintenance of virulence [10,11]. In addition to the members of MGF 505 and 360, four genes appear to be related to virus virulence, the gene coding for thymidine kinase, the NL-S gene (DP71L in BA71V), the UK gene (DP96R in BA71V) and gene 9GL (B119L in BA71V) [12–15]. On the other hand, it has been shown that the deletion of gene EP402R that codes for a protein similar to the cellular CD2 affects viral infection in pigs by delaying the onset of viremia and virus dissemination and also reducing viremia titers [5]. Furthermore, the viral homologue of CD2 shows immunosuppressive activity in vitro [16].

The advent of high throughput genome sequencing has resulted in the rapid increase of the number of ASFV genomes of virulent and nonpathogenic isolates published [2,17–20]. The comparative analysis of the genomic sequences of virulent isolates has provided valuable information about the breadth of ASFV genome sequence and structure variation, along with more precise phylogenetic reconstructions. From the comparison of low virulent and non-virulent isolates valuable targets for the study of the attenuation processes are emerging. The attenuation process is responsible for the appearance of inapparent forms of the disease, in which no clinical signs are observed although viremia and antibodies against ASFV are present [21,22], which difficults the fight against the disease. To date two genome sequences of low virulent isolates, OURT88/3 [2], NH/P68 [20] and one of a non-virulent, BA71V, have been published. OURT88/3 and NH/P68, are low virulent strains isolated in Portugal 20 years apart from different hosts (OURT88/3 from ticks and NH/P68 from swine) whose genomes are almost identical [20]. The genome of the BA71V strain was the first genome sequenced, and to date remains the only genome completely sequenced, including the TIR and hairpin loop sequences that constitute the ends of the genome [1]. In the past 40 years this strain has been used by several research groups as model for ASFV, which has resulted in the accumulation of a large body of experimental information about its biochemical, genetic and morphogenetic behavior. These facts have made of BA71V the ASFV reference strain. BA71V is non infective in swine [FR, personal communication], and was obtained by the adaptation to grow in Vero cells of the isolate BA71, a highly virulent virus obtained from the spleen of an infected animal in Badajoz (Spain) in 1971. Thus, these two isolates, with very different degrees of virulence, are very closely related. We hypothesized that a comparison of the two genomes might help in the identification of virulence factors and viral genes required for replication in the host, and decided to undertake the sequencing of the genome of the virulent isolate BA71. The results that we present here show that the genomes of the strains analyzed, independently of their virulence, are collinear and largely identical, allowing us to identify a relatively small number of changes, responsible for the loss of virulence observed in BA71V. The wealth of information available about the molecular virology of the BA71V strain, and the availability of vectors and methods for its genetic manipulation [23–25], along with the results presented here, make of BA71/BA71V an excellent model for the study of the molecular bases of ASFV virulence.

## Materials And Methods

### Viruses and Cells

The starting material for our study was the sample of BA71 described by Enjuanes et al [26], which has been maintained in our laboratory. The viruses in this sample were passaged 100 times in porcine monocytes [26]. To ensure that we were using a clonal population in our experiments the virus was cloned three times by limited dilution and amplified in porcine alveolar macrophages. After the final amplification, the number of passages in porcine alveolar macrophages of the cloned virus was 10, and the total number of passages from the original sample isolated from the infected pig was 110.

For pig inoculation, extracellular virus was partially purified by sedimentation through a 25% sucrose cushion in PBS for 1 h at 4° in a SW41.Ti Sorvall rotor at 100,000xg. The sediment was resuspended in PBS.

Porcine alveolar macrophages were prepared and cultured as indicated before [27].

### BA71 DNA Sequencing and Analyses

To prepare the templates for sequencing, viral genomic DNA was extracted from semi-purified virus obtained from supernatants of BA71-infected porcine alveolar macrophages and random DNA fragments were obtained by incomplete enzymatic digestion with Tsp509l restriction endonuclease. Fragments ranging from 2 to 3 kbp were cloned into EcoRI-digested pBluescript KS (-) plasmid. These double-stranded DNA templates were sequenced from both ends using dideoxy chain terminator sequencing chemistry, and the reaction products were analyzed on an ABI PRISM 3700 automated DNA sequencer (Applied Biosystems). Base calling was done using the program Phred [28,29]. Contiguous sequences (contigs), were assembled using the program Phrap and edited using the program Consed [30] (http://www.phrap.org; http://Bozeman.mbt.washington.edu/consed/consed.html). The final contig contained 6216 reads and an estimated error rate, as determined by the Consed program, of 0.001 errors per 100 kb. The analysis of this sequence was done using the programs EMBOSS [31], Mauve [32], Geneious [33] and MacVector [31,34].

Four polyG regions at positions 10963, 11194, 11323 and 11518 showed different length in the pBluescript clones sequenced, probably due to replication slippage on these homopolymeric tracts, or to limitations inherent to the sequencing technology used. They occur at intergenic regions, and correspond to the differences 10, 11, 12 and 13 respectively. They were annotated as uncertain in the Genbank record and will not be further discussed.

To allow for a clear understanding of the relationship between the genes of both strains, the nomenclature used for the ORFs of BA71V was maintained. In regions identical in both genomes the BA71V names were conserved, whereas non-identical ORFs were differentiated by the addition of the BA71- prefix. The ORFs in the BA71 genome with no counterpart in BA71V were named following the naming conventions used for the BA71V strain [35] with the addition of the BA71- prefix.

ORF L57L, previously considered a minor ORF in BA71V because of its size, has been now annotated as a major ORF due to its conservation among isolates, to the fact that in BA71 it is a major ORF, and because the variation observed among isolates occurs in the coding sequence, thus not affecting to the putative gene control sequences.

Database searches with ASFV protein sequences were done using the PSI-Blast program [36] at the NCBI server, the jackhammer program at the HMMER web server [37], and the HHblits server [38]. Multiple alignments of protein sequences were done using the Unipro UGENE program [39] using the algorithms Kalign2 [40] and T-Coffee [41].

### Verifcation of the BA71V Sequence

The sequence around the sites in which the BA71 sequence and the BA71V sequence (Genbank accession U18466.1) disagree was determined using as template DNA obtained from the BA71V plasmid library [42] and oligonucleotides located 100 to 200 bases in 5’ of the target sequence. The corrected BA71V sequence has been deposited in GenBank with the accession number U18466.2.

### Virulence of Cloned BA71

To characterize the virulence of the cloned BA71 virus, 5 three-months old male pigs (immunologically mature) of the Duroc x Landrace strain, were housed in the BSL3 facilities at CReSA (Universitat Autònoma de Barcelona). These experiments were performed under the approval of the Ethical and Animal Welfare Committee of the Universitat Autònoma de Barcelona (UAB; Permit number: DMAH-5796). After 5 days of adaptation to the new location, 3 pigs were infected with 10^2^ HAU50 and 2 extra-animals were infected with 10^4^ HAU50 of the BA71 virulent strain grown in pig macrophages and partially purified. All pigs were bled before infection and then intramuscularly inoculated (in the quadriceps) with a volume of 2 ml of PBS containing the corresponding virus dose. The health of the animals including rectal temperatures as well as other ASF-clinical signs were daily recorded, including the time of death for each infected animal. Two pigs infected with the maximal dose of BA71 were found dead at day 6 post-infection. The cause of death was acute ASF. After necropsy pigs showed cyanosis, blood-tinged fluid accumulated in the body cavities, splenomegaly and hemorrhages in the gastrohepatic, mesenteric and renal lymph nodes. Lymph nodes and spleens showed large titres of ASFV (>10^6^ HAU50/g of tissue). Acute and hyperacute ASF is characterized in many occasions by the occurrence of sudden deaths without clinical signs [43], a picture that can be easily reproduced after experimental infection with highly virulence strains as it is BA71. Other pigs were euthanized with an intravenous dose of 140 mg/Kg sodium pentobarbital (Dolethal). The human endpoint was based on clinical criteria previously established in our laboratory for ASFV infection [43]. Briefly, the endpoint criteria decision is based both on quantitative and qualitative indicators. A clinical evaluation scoring system was established to evaluate (in a 0–3 scale) the body (3 different parameters) and the skin conditions, the affection of the respiratory and digestive system and the behavior. Those pigs scoring from 9 to 16 were directly euthanized. Several other qualitative indicators were additionally used. Thus, any animal showing incapacitating prostration, acute respiratory failure symptoms or showing profuse rectal bleeding were immediately euthanized. No additional antipyretic, anti-inflammatory or antibiotic treatment was needed.

### Nucleotide Sequence Accession Number

The genome sequence of the BA71 strain has been deposited in GenBank under accession number KP055815.

## Results and discussion

### Virulence of ASFV BA71 isolate cloned in porcine alveolar macrophages

**Fig 1:**
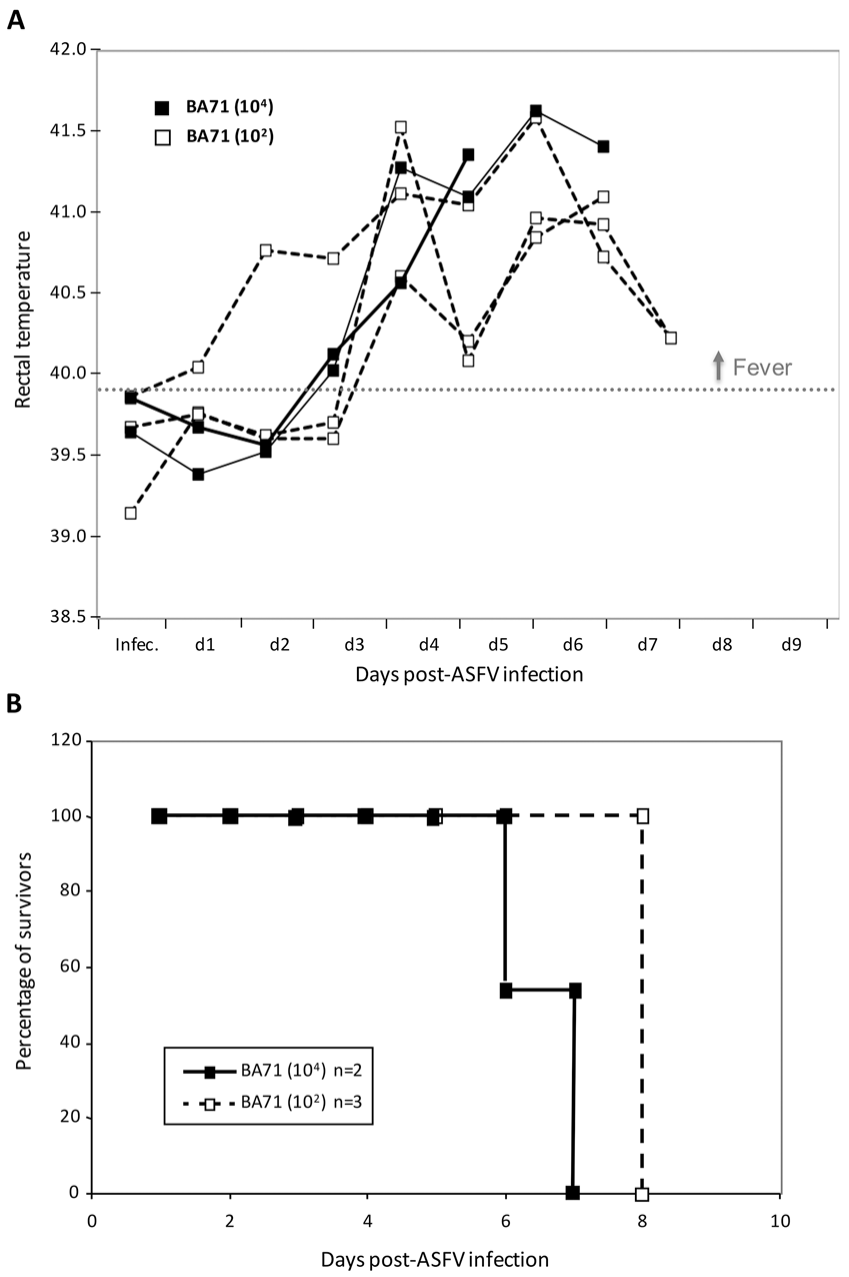
Virulence of ASFV BA71 strain. (A) Rectal temperatures were daily recorded after infection of pigs with 10^2^ or 10^4^ HAU50 of BA71. (B) Survival of pigs after infection with 10^2^ or 10^4^ HAU50 of BA71.

The isolate of ASFV BA71 maintained in our laboratory derives from the virus used for the characterization of this strain by Enjuanes et al. [26], during which it was passaged about one hundred times in swine monocytes. The BA71V strain was derived using the 36th passage of BA71, by adaptation to grow in Vero cells as described [26]. Thus, from the 36th passage on both viruses have diverged: BA71V suffered further passages for the adaptation to growth in Vero cells, and BA71 was subjected to at least 64 additional passages in swine monocytes, to which we must add the 10 additional passages in porcine alveolar macrophages needed for the cloning and expansion procedures of this work. To confirm the virulence of the BA71 isolate after cloning and expansion in porcine alveolar macrophages, pigs were inoculated with semi-purified virus as described in Materials and Methods and ASF clinical signs, including rectal temperature (Fig 1A), were daily recorded, and the time of death for each infected animal registered (Fig 1B).

As expected, *in vivo* inoculation of BA71 was highly pathogenic, causing severe ASF clinical signs, including high fever from day 3 post-challenge to the end of the experiment and independently of the dose used (Fig 1A). All three pigs infected with only 10^2^ HAU50 of BA71 died at day 8 post-infection of acute ASF (Fig 1B).

### The Genome of ASFV BA71 Isolate: Comparison with the BA71V Genome

The sequenced BA71 genome has a length of 180,365 bp, contains 161 open reading frames (ORFs), and spans the complete coding sequences of the BA71V genome, including the ORFs located into the terminal inverted repeats (TIR). Compared with the genome of BA71V, 94 nucleotides at each end remain un-sequenced (the terminal hairpin loops and the first 59 nucleotides of the TIR). This is the most complete sequence of a virulent ASFV strain available, and shows that BA71 possesses the smallest genome of the virulent isolates described so far (Table 1).

**Table 1:** Genomic sequences used in this study.

Isolate	Virulence	Size	Left	Central	Right	Host	Reference	Accesion
**BA71V**	**Avirulent**	**170101**	**29120**	**129780**	**10525**	Vero cell adapted	[1]; this study	NC_001659
**OURT88/3**	**Low**	**171719**	**31274**	**130024**	**9745**	Tick	[44, 2]	AM712240
**NH/P68**	**Low**	**172051**	**31621**	**130010**	**9744**	Pig	[20, 45]	KM262845
**BA71**	**High**	**180365**	**37269**	**129758**	**12662**	Pig	this study; [26]	KP055815
**E75**	**High**	**181187**	**39758**	**129733**	**11020**	Pig	[18, 46]	FN557520
**Benin97/1**	**High**	**182284**	**40454**	**129911**	**11243**	Pig	[2, 47]	AM712239
**Lisbon60**	**High**	**182362**	**40803**	**129633**	**11250**	Pig	[20, 45]	KM262844
**Tengani62**	**High**	**185689**	**41386**	**129715**	**13912**	Pig	[48, 49]	AY261364
**Malawi Lil-20/1**	**High**	**187612**	**43504**	**132119**	**11313**	Tick	[50, 51]	AY261361
**Georgia2007/1**	**High**	**189344**	**46308**	**129430**	**12930**	Pig	[52, 19]	FR682468
**Pretoriuskop/96/4**	**High**	**190324**	**46921**	**129764**	**12963**	Tick	[53, 54]	AY261363
**Kenya 1950**	**High**	**193886**	**49214**	**131070**	**12899**	Pig	[53, 17]	AY261360

The table shows general information about the genomic sequences of the ASFV strains used in this study. Left and right indicates the size of the left and right genome variable regions. The left variable region includes the nucleotides from the 5’- end of the genome until gene A224L, whereas the right variable region includes the nucleotides from the 3’- end of the genome until gene DP238L.

**Fig 2:**
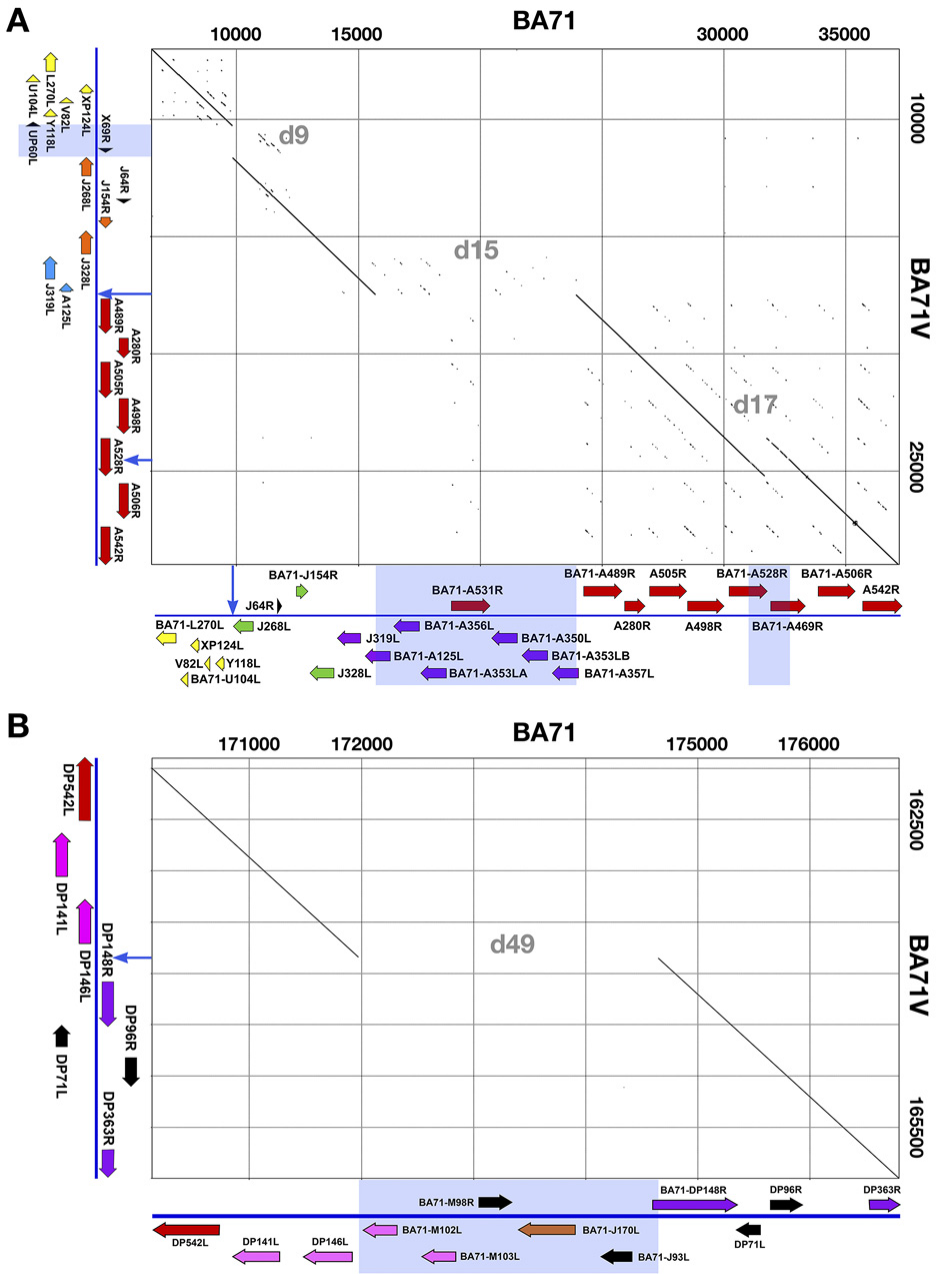
5’- and 3’- ends of the ASFV BA71 and BA71V genomes. The figure shows dot plot comparisons of the 5’- (A) and 3’-end (B) variable regions. For each of the four large deletions observed between the genomes, the deleted region is indicated by a light blue box in the corresponding axis and the position where the deletion occurred is indicated by an arrow in the opposite axis. ORFs are indicated by arrows, and the different groups of ORFs are identified by colors: MGF 360, violet; MGF 110, yellow; MGF 300, green; MGF 505, red; MGF 100, rose; p22 family, gray, other ORFs, black.

BA71 and BA71V genomes are collinear and largely identical. The identity is interrupted 69 times by 37 point mutations and 32 insertions or deletions. Eighty of the 170 kbp of the sequence of BA71V genome were obtained as a collage of small sequencing projects with very different techniques, including chemical sequencing [1]. Given the high quality of the BA71 sequence and the extent of the identity between both genomes, we thought possible that some of these differences were due to errors in the sequence of the BA71V genome. To answer this question, we verified the sequence of BA71V in the positions where it differs from BA71 in the plasmid library used for the determination of the original BA71V sequence [42]. The errors detected are summarized in S1 Table. The corrections introduced in the sequence of BA71V do not alter the total number of nucleotides but modify some genome features. The sequence of the left TIR, and the ORFs KP360L, KP362L, J268L, J104L, J182L, C122R and DP311R are altered. The sequence of the encoded protein is modified for KP360L, J268L and DP311R. In the corrected genome, the sequence of ORFs J104L and J182L is fused into a larger ORF, J328L. This ORF is a member of the MGF 300, orthologous to MGF300-4L, and is present with a similar size in all the viral genomes sequenced to date. The correction at position 67449 changes the size of the involved ORF (C122R) to 105 amino acids, the size of the orthologous protein in other ASFV genomes. According to the naming conventions for the BA71V strain the ORF is now labeled as C105R. The correction of the error at position 13543/13544 results in the increment to 64 amino acids of a previously considered minor (non-annotated) ORF. Orthologues to this ORF, J64R, are present in the genomes of the European isolates (E75, Lisbon60, OURT88/3, NH/P68, annotated as X64R), and in some African isolates (Pretoriuskop/96/4 and Benin97/1, although not annotated). Interestingly, although this ORF does not show any detectable similarity to proteins in the databases, it is remarkably similar to the ASFV ORF X69R (S1 Fig). After the corrections introduced in the sequence of the BA71V genome, the alignment between the BA71 and BA71V genomes is interrupted 52 times by 30 point mutations and 22 deletions or insertions. These differences can be grouped in four classes according to its nature: (i) Large deletions in the variable regions of the genome (1354–8238 nts, Fig 2, Table 2), likely originated by misalignment during replication or by recombination between the highly similar sequences of the multigene families; (ii) Small deletions or duplications (10–408 nts, Table 3) produced by variations in the number of tandem repeats by slipped-strand mispairing during replication [55]; (iii) Variations of one or two nucleotides in polynucleotide tracks (Table 4) produced by polymerase slippage during replication and (iv) Point mutations (Table 5), produced during genome replication or repair. We will refer to the different changes by its ordinal number from 5’ to 3’ of the genome, which is indicated in the first column of Tables 2–5.

**Table 2:** Differences between BA71 and BA71V: Large deletions/insertions.

Difference Number	BA71		BA71V	
	Boundaries [nts deleted in BA71V]	Affected genes	Boundaries [nts deleted in BA71]	Affected genes
9	9840–9857 [18]		10310–11663 [1354]	UP60L, X69R
15	15701–23938 [8238]	BA71-A125L, BA71-A356L, BA71-A353A, BA71-A531R, BA71-A350L, BA71-A353LB, BA71-A357L	17496/17497	A125L
17	31057–32763 [1707]	BA71-A528R, BA71-A469R	24613/24614	A528R
49	171992–174660 [2669]	BA71-M103L, BA71-M98R, BA71-J170L, BA71-J93L, BA71-DP148R	163862/163863	DP148R

The positions in the genome of BA71 and BA71V of the sequences affected are indicated along with the size of the region not present in either of the genomes, in brackets. The genes affected by theses differences are also indicated.

**Table 3:** Differences between BA71 and BA71V: Variations in the number or in the sequence of tandem repeats.

Difference Number	BA71 boundaries [nts deleted in BA71V]	BA71V boundaries [nts deleted in BA71]	Repetitions involved in the variation	Feature affected
1	827/828	922–1329 [408]	12 x 33 ntds	Left TIR
2	1458/1459	1961–1993 [33]	1 x 33 ntds	KP86R/KP93L
4	5127–5156 [30]	5661/5662	2 x 15 ntds	L57L
7	7631–7665 [35]	8135/8136	2 x 18 ntds	L270L 3’UTR
21	38215/38216	30067–30076 [10]	1 x 10 ntds	
31	79249/79250	71111–71123 [13]	1 x 13 ntds	
33	93008–93045	84882–84919	4 x 12 ntds	B602L
51	178939/178940	168141–168173 [33]	1 x 33 ntds	DP86L/DP93R
52	179920/179921	169155–169562 [408]	12 x 33 ntds	Right TIR

The positions in the genome of BA71 and BA71V of the sequences affected are indicated along with the number of repeats involved in the variation, and the identity of the genomic feature affected by the change. The size of the region not present in either of the genomes is indicated in brackets. In the case of difference 33, the highly variable central region located in ORF B602L, 4 of the 29 repeats of 12 nucleotides that compose this region have different sequence but there is no variation in the number of nucleotides.

**Table 4:** Differences between BA71 and BA71V: changes in polynucleotide sequences.

Difference number	BA71	BA71V	Polynucleotide	ORF
16	25610 [1]	19167/19168	Poly A	A489R
18	33141/33142	24992 [1]	Poly T	A528R
35	116146 [1]	108019/108120	Poly T	CP204L
48	168787–168788 [2]	160659/160660	Poly A	DP311R/DP63R
50	177923 [1]	167124/167125	Poly A	

The position of the differences in each genome is indicated along with the size of the change, in brackets. The nature of the polynucleotide and the ORF affected, if any, are also indicated.

**Table 5:** Differences between BA71 and BA71V: Point mutations.

Difference Number	BA71	BA71V	ORF in BA71V	Function	Mutation
3	3312 [C]	3847 [T]	KP362L	MGF 360	Silent
5	6344 [T]	6849 [C]	L356L	MGF 360	T69A*
6	7377 [C]	7882 [T]	L270L	MGF 110	D71N
8	7832 [T]	8302 [C]	U104L	MGF 110	Y78C
14	12928 [T]	14724 [A]	J154R	MGF 300	C155STOP
19	34010 [T]	25861 [A]	A506R	MGF 505	I110N
20	38025 [T]	29876 [C]	NO ORF		
22	38712 [A]	30573 [G]	A240L	Thymidylate kinase	STOP210Q*
23	42115 [T]	33976 [C]	A238L	vIkB	Silent
24	47403 [T]	39264 [C]	F334L	Ribonucleotide reductase	M207V
25	60279 [A]	52140 [G]	EP1242L	RNA pol sub.2	L327P
26	65186 [A]	57047 [G]	EP402R	vCD2	K269E*
27	66982 [A]	58843 [C]	M1249L	Unknown	Y1209D
28	68556 [T]	60417 [C]	M1249L	Unknown	D684G
29	71050 [C]	62911 [T]	M448R	Unknown	Silent
30	76144 [C]	68005 [T]	C275L	Unknown	E227K
32	85709 [C]	77583 [A]	B962L	Helicase	Q125H
34	104885 [G]	96759 [A]	G1211R	DNA polymerase	R123Q
36	118468 [T]	110341 [C]	CP80R	RNA pol sub.10	L42P*
37	120840 [A]	112713 [G]	NP1450L	RNA pol sub.1	Silent
38	122720 [C]	114593 [T]	NP1450L	RNA pol sub.1	E672K
39	126852 [T]	118725 [C]	NP868R	Guanylyltransferase	F238S*
40	128046 [G]	119919 [A]	NP868R	Guanylyltransferase	G636D*
41	129504 [C]	121377 [G]	D250R	Nudix hydrolase	I234M
42	138196 [C]	130069 [T]	S273R	Protease	Silent
43	148182 [A]	140055 [G]	R298L	Protein kinase	Silent
44	152823 [A]	144696 [G]	QP383R	NifS-like	Silent
45	153664 [A]	145537 [G]	E183L	p54	V143A*
46	159308 [G]	151181 [A]	E120R	p14.5	E89K
47	160575 [G]	152448 [A]	E111R	Unknown	D83N

The positions of the point mutations in both genomes are indicated along with the nature of the nucleotides at each position, in brackets. The ORF implicated, its known function and the mutation produced in the protein sequence are also indicated. The asterisk indicates that a comparison with the sequences of other European isolates shows that the mutation has occurred in the virulent BA71 isolate.

#### Large Deletions

The comparison of the genomes indicates the presence of four large deletions in left and right variable regions (Table 2, Fig 2). Three of them, difference 9 (d9), d17 and d49, occur in positions where changes in the genetic organization are frequently observed between strains. Such rearrangements, found in virulent and attenuated viruses, are largely responsible for the variations in genome length observed between strains.

**Fig 3:**
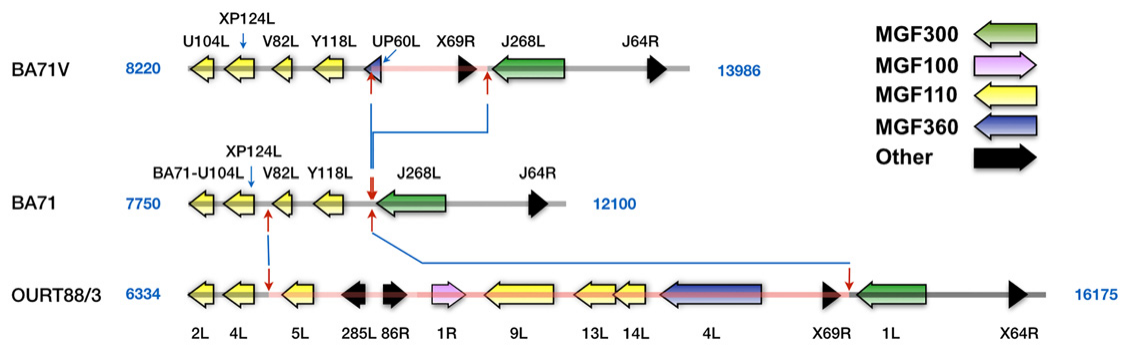
Comparison of the genomic structure of BA71 with BA71V and OURT88/3 around difference 9. The figure shows a representation to scale of the genomes of BA71, BA71V and OURT88/3 around the position of BA71-BA71V difference 9 (the exact positions are indicated for each of the genomes). Red arrows delimit non-identical regions, which are indicated by a central red line in the BA71V and OURT88/3 genomes. Blue lines connect arrows at equivalent positions. The different groups of ORFs are identified by colors as indicated in the figure.

The first large deletion, d9, corresponds to a difference of 1354 nts in the MGF110 region of the LVR of the genomes (Fig 3, S2 Fig). The different sequence includes two genes, UP60L, member of the MGF360, and X69R, a small protein of unknown function, similar to J64R as indicated previously (S1 Fig), which is maintained in the genome of all sequenced viruses. These sequences are deleted in the virulent BA71 but conserved in the attenuated BA71V, thus very unlikely related to virulence. This deletion in the BA71 genome probably occurred during the passages given to BA71 after the sample used to generate BA71V was separated [26]. This region varies widely among isolates, without regard to their virulence, due principally to changes in the number of genes of MGF100, -110 and -360. Interestingly, BA71 possesses the smaller genetic complement in this region, only 6 genes. This compares with the 16 genes present in the Georgia2007/1 isolate in the same region.

**Fig 4:**
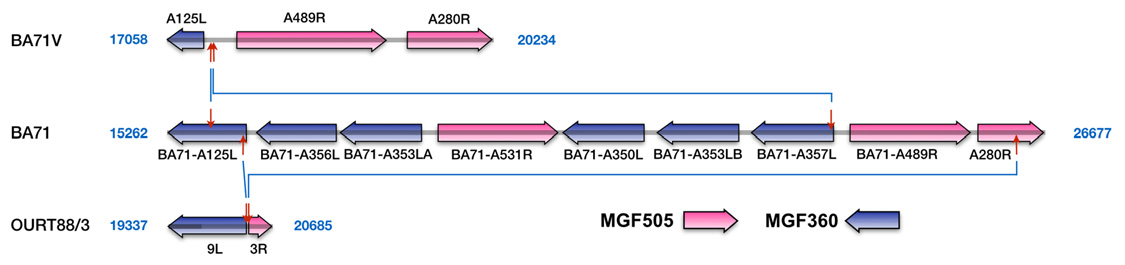
Comparison of the genomic structure of BA71 with BA71V and OURT88/3 around difference 15. The figure shows a representation to scale of the genomes of BA71, BA71V and OURT88/3 around the position of BA71-BA71V difference 15 (the exact positions are indicated for each of the genomes). Red arrows delimit non-identical regions. Blue lines connect arrows at equivalent positions. The different groups of ORFs are identified by colors as indicated in the figure.

In contrast with the other differences, d15 (Table 2, Fig 4, S3 Fig), the largest deletion, occurs only in attenuated strains, thus all the sequenced genomes of virulent viruses possess a conserved genomic structure in this area, whereas all the attenuated non-recombinant viruses possess deletions in this region [9–11,19,56]. Experimental results are in agreement with the existence of a link between attenuation and the deletion of MGF360 and -505 in this region [2,10,19,20]. There is less agreement, however, about a possible role for the genes encoded in this region and the ability of the virus to replicate in macrophages. Thus, whereas in the attenuated BA71V replication in macrophages could be rescued by the insertion of three of the MGF360 genes of his region [9], OURT88/3, which possesses a larger deletion in this region than BA71V (Fig 4), was shown to efficiently replicate in macrophages [57]. Also, the deletion mutant E70∆NL-S, that did not show any defect on macrophage replication [14], was later shown to have a deletion in this region very similar to that of BA71V [10]. Finally, conflicting evidence has been shown about the ability of the strain BA71V to grow in macrophages [9,58,59].

Interestingly, a deletion mutant of the Pretoriuskop/96/4 isolate, Prt4∆3-C2, that lacks genes orthologous to BA71-A125L, BA71-A356L and BA71-A356LA shows a 100- to 1000- fold reduction on viral replication in infected ticks [56], suggesting an impairment of the ability of BA71V to replicate in ticks. However, the extent of this impairment probably depends on the genomic context since a similar deletion is observed in OURT88/3, which was isolated from ticks [44].

**Fig 5:**
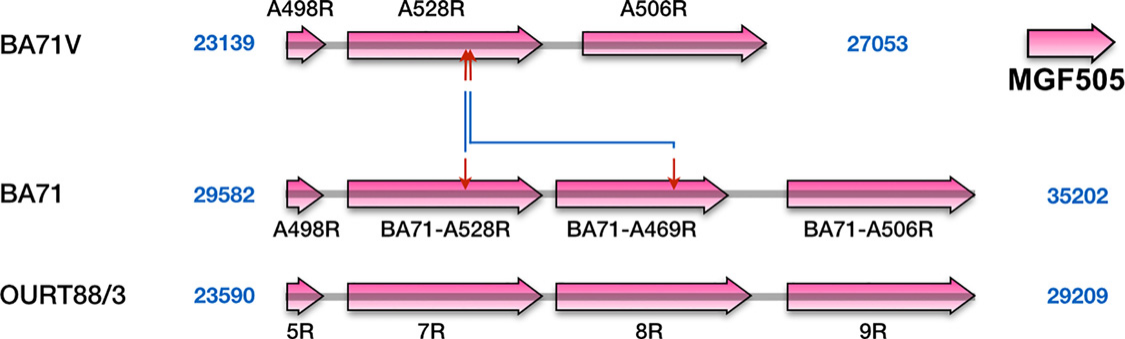
Comparison of the genomic structure of BA71 with BA71V and OURT88/3 around difference 17. The figure shows a representation to scale of the genomes of BA71, BA71V and OURT88/3 around the position of BA71-BA71V difference 17 (the exact positions are indicated for each of the genomes). Red arrows delimit non-identical regions. Blue lines connect arrows at equivalent positions. The ORFs are members of MGF 505.

The third large deletion, d17 (Table 2, Fig 5, S4 Fig), appears to be the product of a recombination event between two members of the BA71 MGF 505, BA71-A528R and BA71-A469R. In BA71V the gene A528R corresponds to the fusion of the 5’-half of gene BA71-A528R (orthologous to MGF 505-7R) with the 3’-half of gene BA71-A469R (orthologous to MGF 505-8R). A similar genetic structure than that of BA71V is observed in the virulent Malawi Lil-20/1 and Kenya 1950 isolates, whereas the attenuated OURT88/3 possesses a structure similar to that of BA71, or Lisbon60; thus it seems unlikely that this deletion is related to virulence.

**Fig 6:**
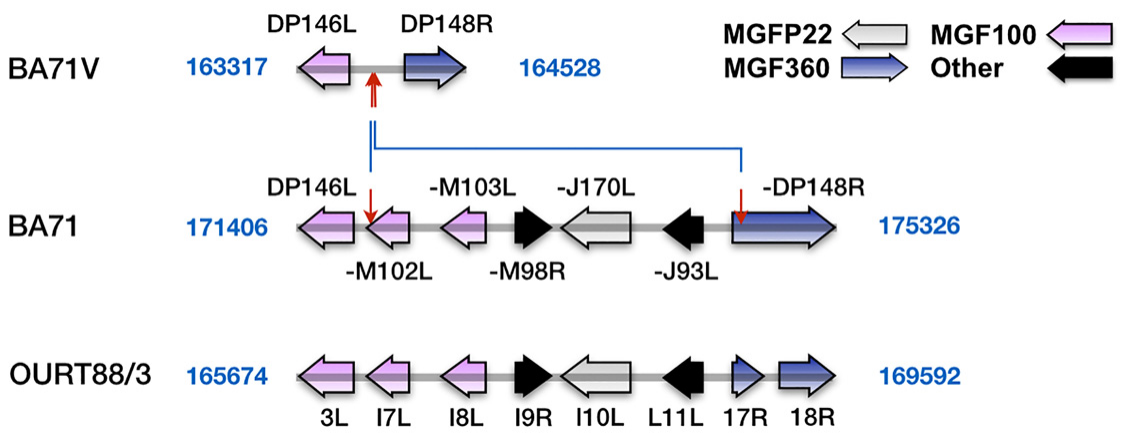
Comparison of the genomic structure of BA71 with BA71V and OURT88/3 around difference 49. The figure shows a representation to scale of the genomes of BA71, BA71V and OURT88/3 around the position of BA71-BA71V difference 49 (the exact positions are indicated for each of the genomes). Red arrows delimit non-identical regions. Blue lines connect arrows at equivalent positions. The different groups of ORFs are identified by colors as shown in the figure. The dash at the beginning of the names of some ORFs indicates where the prefix BA71- has been removed to avoid clutter.

Finally, the fourth large deletion, d49, in BA71V affects the 3’-end of the genome (Table 2, Fig 6, S5 Fig) in a region that contains members of the MGF100, P22 and MGF360 multigene families. This region is variable among virulent isolates but all of them maintain the minimal set of genes found in BA71, Lisbon60 or Georgia2007/1. This set includes two members of the multigene family 100 (BA71-M102L, -M103L), a member of the MGF P22 (BA71-J170L), the gene BA71-M98R, which is highly similar to the ASFV gene L83L (S1 Fig) but has no other detectable similarities to database sequences, and the gene BA71-J93L, which also has no known similarities. Deletions in this region are only observed in BA71V and in the Vero cell adapted Georgia G/VP110 strain [19], suggesting that although the deletion in d15 appears to be the main cause of the attenuation, this deletion may contribute to the extent of attenuation found in these highly attenuated strains.

#### Variation In The Number Or Sequence Of Tandem Repeats

Tandem repeats are highly dynamic structures with a probability of mutation orders of magnitude higher than that of point mutations [60]. However, changes of this nature, especially in the highly compact ASFV genome, often result in frameshift mutations that will be subjected to a strong purifying selection. Thus, although in the BA71V genome there are 43 regions of tandem repeats with an ETANDEM [31] score higher than 20 (not shown), the comparison of BA71 and BA71V genomes indicates that there are fewer changes in tandem repeats than point mutations, and the majority occur at the intergenic regions or in the TIR (Table 3). The core of the TIR, which in BA71 is formed by 22 tandem repeats of a 34 nucleotides sequence (RDT34) [61] is expanded to 34 repetitions in BA71V. The importance of this change is unknown since very little is known about the relevance of this structure for ASFV replication. Two of the other intragenic variations in tandem repeats appear not to be involved in coding regions or in regions related to the control of gene expression (d21, d31). However, d7 occurs in the 3’ untranslated region of the MGF110 gene L270L messenger mRNA [62], a region that could be potentially important for the control of the mRNA stability, as has been shown for other systems [63].

A remarkable exception to the potentially deleterious nature of the variation in tandem repeats occurs when the repeated sequence maintains the reading frame of the protein, and the protein structure is tolerant to the expansion and contraction of the repeated block. In bacteria and *S*. *cerevisiae* this type of proteins frequently encodes adhesion factors, and the alteration in the number of repeats within the block has been related to the adaptation of the microorganism to changes on environmental conditions and evasion of host immune system [55]. The comparison of BA71 and BA71V genome sequence shows the presence of three ORFs with differences in the number or the sequence of tandem repeats between these isolates (Table 3). The region of tandem repeats found in the coding sequence of protein B602L possesses a high variability between ASFV isolates [5,64], produced by changes in the number of repetitions of a 12 nts sequence, thus maintaining the reading frame of the encoded protein. B602L is an essential protein whose function is to chaperone the folding of the major structural protein p72 [65,66], and that appears to be highly tolerant to the expansion and contraction of the four-amino acid repeat domain produced by this tandem repetition. Interestingly, there is no change in the number of tandem repeats between BA71 and BA71V, but 4 out of the 29 repeats that compose the region have changes in its sequence (not shown).

This type of variation is also observed in L57L (S6 Fig), an ORF of unknown function that was not previously annotated in BA71V (see Materials and methods section). This ORF, present in all the isolates whose genome has been sequenced (annotated as L60L, or unannotated [in Malawi Lil20]), shows a variation in size that ranges from 93 amino acids in Malawi Lil-20/1 to 52 of the orthologous gene in the Georgia2007/1 strain. This variability is produced by the change in the number of a 15 nts repetition block located in the 3’-end of the ORF. Two of the 6 repeats of 15 nt present in the BA71 ORF are lost in BA71V.

The third block of tandem repeats in a coding sequence occurs in the partially overlapping ORFs found in the TIR (Table 3) [64]. In BA71V there is a block of three RTD33 direct tandem repeats [61] that is originated from the duplication of one of the two RDT33 repeats found in BA71. Although the TIR region is not present in all the ASFV genome sequences present in the databases, all the European isolates whose genome sequences include this region maintain the BA71 structure. This change affects two ASFV genes of unknown function that in some strains are perfectly repeated, although inverted, at both ends of the genome (KP86R/DP86L, KP93L/DP93R), and that possess in BA71V a repetition of 11 amino acids close to the c-terminus of the encoded protein.

#### Variation in the length of polynucleotide tracks

Variations in polynucleotide tracks are produced by the classical Streisinger template-slippage mechanism [67], and often result in frameshift mutations when they occur in a coding region. Probably due to the compact nature of the ASFV genome, the majority of the mutations of this kind are found within ORFs; however, given the impact of frameshift mutations, this type of mutations is found mainly in members of the multigene families, which probably provides some level of functional redundancy, and always occur close to the c-terminus of the encoded protein, thus reducing the extent of the mutated sequence (Table 4).

The poly-A track that begins at position 168649 in BA71V is one of the longest in the ASFV genome, making it mutation-prone. It is located in the region of overlap between the ORFs DP311R and DP63R, thus changes in its sequence affect simultaneously to this two ORFs. We can distinguish three types of genomes according to the length of this poly-A track and its effects in the length of the DP311R and DP63R and its orthologous genes. In BA71, the presence of a 13-nucleotide-long poly-A track gives rise to a 352 amino acid-long BA71-DP311R gene, and a 21 amino acid-long (minor) DP63R. A similar structure is observed in the genome of the Kenya 1950, Malawi Lil-20/1 and Pretoriuskop/96/4 strains. The deletion of a single nucleotide in the poly-A track produces a shift in both reading frames that results in a premature termination in the case of DP311R, reducing its length to 310 amino acids, and in an extension to 23 amino acids in the, still minor, ORF orthologous to DP63R. This occurs in the genomes of the Benin97/1, Georgia2007/1 and Lisbon60 strains. It is interesting to notice that the attenuated Georgia/VP110 has an identical structure according to Krug et al. [19]. Finally, the shift in frames produced by the deletion of two nucleotides produces a 311 amino acid-long DP311R ORF, whereas for DP63R it results in an in-frame fusion with the sequence that in the previous cases would correspond to the 48 amino acids in the 3’ region of BA71-DP311R, extending the MGF DP63R to 63 amino acids. This occurs in the case of the virulent strain E75 and in the attenuated strains BA71V, OURT88/3 and NH/P68 (although DP63R is not annotated in these two latest genomes), thus it is very unlikely related to attenuation.

The deletion of the last base in a 10-T track on the penultimate codon of the CP204L ORF produces in BA71V a C-terminal extension of 10 amino acids in the phosphoprotein p32, an early, highly-immunogenic, ASFV polypeptide of unknown function, that forms 220 kDa homo-oligomers in the cytoplasm of the infected cell [68]. This ORF suffers numerous changes among isolates, and variations in this poly-T track contributes to those changes in Georgia2007/1, BA71V and NH/P68. Interestingly, BA71V and NH/P68, both attenuated, share the same sequence for this protein, whereas the attenuated OURT88/3 shares identical sequence to the virulent viruses, except for the Georgia2007/1 strain, where a deletion of two T bases produces a unique 8-amino acids extension of different sequence.

The deletion of a base in the 6-A track on the 3’-end of the gene orthologous to BA71-A489R gene in BA71V provokes a change in the reading frame, which shortens the encoded protein from 526 amino acids in BA71 to 489 in BA71V, including a 9-residues long dissimilar sequence. Interestingly, a similar mutation in the virulent E75 strain, highly similar to BA71, produces a shorter version of the orthologous gene, MGF505-2R, without effect on the apparent virulence.

#### Common Orfs in BA71 and BA71V: Conservation and Variation

BA71 encodes 161 major ORFs, whereas BA71V encodes 152 of which 149 are common to both genomes. Of these, 118 are identical and thus are not involved in the changes in virulence observed between the two strains. However, there are alterations in the sequence of 29 of the common ORFs that could be related to these phenotypical differences. These changes are due to deletions/insertions, as indicated above, or point mutations, as shown in Table 5. Comparison of the sequence of the point mutations with the genome of other virulent isolates indicates that, in some cases, the point mutations have likely occurred in the virulent isolate BA71, and not in the genome of the BA71V. Thus, for example, the thymidylate kinase involved in DNA metabolism and encoded by gene A240L is 240 amino acids long in all the sequenced genomes, attenuated or virulent, with the exception of BA71, where it is 31 amino acids shorter due to a mutation in position 38712 that produces a premature in-frame stop codon. There are a total of 7 positions which have the same sequence in all the European isolates, including BA71V, but which are mutated in BA71, and that likely do not have any relation with the changes observed in the virulence (indicated in Table 5 with an asterisk). Taking into account the changes in non-coding sequences, silent changes, and the mutations that likely occurred in BA71, there are 15 point mutations (that affect 14 genes) that could contribute to the phenotypic changes between strains. Interestingly, M1249L a gene of unknown function, which is mutated twice in BA71V, is the only gene mutated in the adaptation processes of both BA71 and Georgia 2007/1 viruses [19].

### Comparison of Protein Sequences

Comparison of BA71 protein sequences to protein sequence databases revealed previously undescribed similarities.

Thus, the protein encoded by gene C105R presents similarity with the archaebacterial transcription factor S (TFS) and the 15 kDa RNA polymerase subunit M, that belong to the archaeal rpoM/eukaryotic RPA12/RPB9/RPC11 RNA polymerase family (S7 Fig). The polypeptides in the archaeal members of this family can be stably integrated into the RNA polymerase as a subunit (subunit M) or act as an exogenous transcription factor (TFS), depending on the archaeal system [69,70] and are related to subunits RPA12, RPB9 and RPC11 of eukaryotic RNA polymerases I, II and III, respectively. Interestingly, the RPB9 and RPC11 subunits are related to the transcript cleavage factor TFIIS and mediate the RNA cleavage activity of their respective RNA polymerases [71,72]. Similarly, the corresponding archaeal polypeptide stimulates the transcript cleavage activity of the archaeal RNA polymerase [73,74]. This suggests that the ASFV C105R protein product may play a similar function in transcription. However, in relation to this it should be noted that the virus also codes for a TFIIS homologue, the product of gene I243L [35], raising the question of whether these two putative cleavage factors play distinct roles in transcription. Interestingly, this similarity is shared also by the protein YP_009041600 of Faustovirus E12, the prototype isolate of the Faustovirus, a new giant virus lineage that infects *Vermamoeba vermiformis*, a protist living in humans and its environments [75]. This virus, with a circular double-stranded genome of 466 kbp shows the presence of several genes that are most similar to ASFV genes. The similarities appear to be restricted to genes involved in genome replication, transcription and repair, as well as some structural genes, including very distinct ASFV genes like the two polyproteins pp220 and pp62 whose proteolytic products construct the core-shell of the ASFV mature particle, as well as the protease pS273R which processes those polyproteins. Given that the previously identified ASFV-like sequences from metagenomic studies [76, 77] have been shown to be more related to Faustovirus than to ASFV by Reteno et al. [75], Faustovirus appears to be the closest relative to ASFV.

New potential roles in the regulation of the virus-cell interaction have also been identified for some ASFV genes. Gene I226R encodes a protein with similarity to the bacterial transcriptional regulatory factor DpiA, and the chemotaxis proteins CheY, response regulator proteins of bacterial two-component systems (S8A Fig) [78]. Two-component systems constitute the signal transduction mechanism prevalent in prokaryotes, but also present in plants, fungi, diatoms, and slime molds. These simple signaling systems regulate most aspects of bacterial life, including responses to almost all changes in the environment of a bacterium [79]. The canonical bacterial two-component system is formed by two proteins: a sensor histidine kinase and a response regulator. The similarity of I226R appears to be higher to the amino-terminal half of the response regulator proteins, where the signal receiver domain is located. Interestingly, the protein encoded by gene E423R appears to be related to the signal transduction protein histidine kinases that function as sensor partners in the two-component signaling pathways, catalyzing the phosphorylation of transcription regulators like DpiA in response to environmental stimuli (S8B Fig) [80]. Eukaryotic two-component systems have experimented a vast divergence from the canonical bacterial form, being adapted to the specialized needs of eukaryotes [81], which makes it very interesting to determine the role of this putative two-component system in ASFV biology.

Furthermore, the C-terminal half of the protein encoded by BA71-A531R, one of the genes of the MGF505 present in BA71 but deleted in BA71V, is similar to the sequence of protein K145R, and this similarity is shared by its orthologues in other virulent isolates (MGF505-1R; S9 Fig). The similarity spans the complete sequence of protein K145R, which indicates that K145R could be considered a distant member of this multigene family. This is somewhat unusual since MGF members are found at the ends of the genome, while gene K145R is located within the central region, but a similar situation occurs with protein A276R, member of the MGF360, which is located also outside the variable regions of the end of the genome.

## Concluding Remarks

Only 28 of the 52 differences found in ASFV BA71V represent a loss or change of genetic information with respect to BA71 and can be linked to the loss in virulence on BA71V.

The contribution of each of these differences to the change of total genetic information is very dissimilar. Experimental data, as well as its presence exclusively in attenuated strains indicates that the main contribution to the attenuation in BA71V is probably due to the deletion in d15, a deletion in the 5’-end of the genome of 8238 nts, that affects six FAM360 members (BA71-A125L, -A356L, -A353A, -A350L, -A353LB, -A357L) and one FAM505 member (BA71-A531R). However, OURT88/3 and NH/P68 possess a larger deletion in the same region, which produces a considerably smaller attenuation, thus suggesting the contribution of additional factors to BA71V attenuation. Its presence exclusively in BA71V and in the Georgia G/VP110 strain [19], both of which are highly attenuated strains, suggests an important role for d49 in the extreme attenuation of these strains. This deletion of 2669 nts in the 3’-end of the genome affects two members of the MGF100 (BA71-M103L, -M102L), a member of the MGF360 (BA71-DP148R), one member of the P22 family (BA71-J170L) and two genes of unknown function (BA71V-M98R, -J93L). However, there are numerous additional changes whose contribution could only be ascertained by means of experimental data.

It is worth noting that the sequences of genes NL-S (DP71L), UK DP96R), 9GL (B119L) and that of the thymidine kinase gene (K196R), described as virulence factors of ASFV, do not vary, and can be ruled out as factors responsible for the loss of virulence in the BA71/BA71V model.

The data reported here should facilitate the identification of factors that determine the virulence of ASFV by generating mutant viruses in the regions of discontinuities that give rise to the loss of virulence in BA71V. The identification of virulence genes in BA71 will provide information on the regions that should be deleted/mutated to attenuate this and other virulent isolates for vaccine development. In addition, the new information obtained on ASFV proteins from the database searches provides novel research targets for future studies.

## Supporting Information

S1 Fig A. Sequence conservation between the amino acid sequences of ASFV ORFs X69R and J64R. Alignment of the amino acid sequences of BA71V ORFs X69R and J64R and its orthologous in other strains. **B. Sequence conservation between the amino acid sequences of ASFV ORFs L83L and M98R.** Alignment of the amino acid sequences of BA71 ORFs L83L and M98R and its orthologous in other strains. Solid and shaded backgrounds indicate identical or similar amino acids, respectively.
(TIF)

S2 Fig Comparison of the genomic structure of BA71 with other ASFV virulent isolates around difference 9. The figure shows a representation to scale of the genomes of BA71, Georgia 2007/1, Lisbon 60, Malawi Lil-20/1 and Pretoriuskop/96/4 around the position of BA71-BA71V difference 9 (the exact positions are indicated for each of the genomes). Red arrows delimit regions not present in the genome of BA71, indicated by a central red line on the genome of the virulent isolates. The different groups of ORFs are identified by colors as shown in the figure.
(TIF)

S3 Fig Comparison of the genomic structure of BA71 with other ASFV virulent isolates around difference 15. The figure shows a representation to scale of the genomes of BA71, Georgia 2007/1, Kenya 1950, Lisbon 60, Malawi Lil-20/1 and Pretoriuskop/96/4 around the position of BA71-BA71V difference 15 (the exact positions are indicated for each of the genomes). The different groups of ORFs are identified by colors as shown in the figure.
(TIF)

S4 Fig Comparison of the genomic structure of BA71 with other ASFV virulent isolates around difference 17. The figure shows a representation to scale of the genomes of BA71, Georgia 2007/1, Lisbon 60, Pretoriuskop/96/4, Malawi Lil-20/1 and Kenya 1950 around the position of BA71-BA71V difference 17 (the exact positions are indicated for each of the genomes). The ORFs are members of MGF 505.
(TIF)

S5 Fig Comparison of the genomic structure of BA71 with other ASFV virulent isolates around difference 49. The figure shows a representation to scale of the genomes of BA71, Georgia 2007/1, Lisbon 60, Malawi Lil-20/1 and Kenya 1950 around the position of BA71-BA71V difference 49 (the exact positions are indicated for each of the genomes). Red arrows delimit regions not present in the genome of BA71, indicated by a central red line on the genome of the virulent isolates. The different groups of ORFs are identified by colors as shown in the figure. The dash at the beginning of the names of some ORFs indicates where the prefix BA71- has been removed to avoid clutter.
(TIF)

S6 Fig Tandem repeats in ASFV ORF L57L. Alignment of the amino acid sequence of BA71V ORF L57L and its orthologous in other strains. The sequence of the orthologous in Lisbon 60, NH/P68, E75 and Benin97/1 is identical to that of OURT88/3. Solid and shaded backgrounds delineate the tandem repetitions.
(TIF)

S7 Fig Similarity of ASFV ORF C105R with members of the RNA polymerase subunit M family and Faustovirus protein YC_009041600. Solid and shaded backgrounds indicate identical or similar amino acids, respectively.
(TIF)

S8 Fig Two-component signaling pathway. A. Comparison of the amino acid sequences of ASFV ORF I226R and the transcription regulatory factor DpiA from *Lactobacillus casei* (strain Bl23) and several cheY chemotaxis proteins. The solid line indicates the region encoding the signal receiver domain. B. Comparison of the amino acid sequences of ASFV ORF E423R and several protein histidine kinase from Clostridium whose accession numbers are indicated in the alignment. Solid and shaded backgrounds indicate identical or similar amino acids, respectively.
(TIF)

S9 Fig Similarity of ASFV K145R with members of the multigene family 505. Alignment of the amino acid sequences of ASFV ORF K145R and the MGF 505 member BA71-A531R and its orthologous (1R) in other ASFV genomes. Solid and shaded backgrounds indicate identical or similar amino acids, respectively.
(TIF)

S1 Table Errors corrected in the sequence of BA71V. The table indicates the position of the changed nucleotides in the original BA71V sequence (Column U18466.1), the nature of the correction, the number of nucleotides lost or gained in each change, and the features affected by the correction.
(DOCX)
