# Supplementary figures and images for: Genome Sequence of African Swine Fever Virus BA71, the Virulent Parental Strain of the Nonpathogenic and Tissue-Culture Adapted BA71V
Source: PLoS One. 2015 Nov 30;10(11):e0142889. doi: 10.1371/journal.pone.0142889 (PMC4664411; doi:10.1371/journal.pone.0142889)

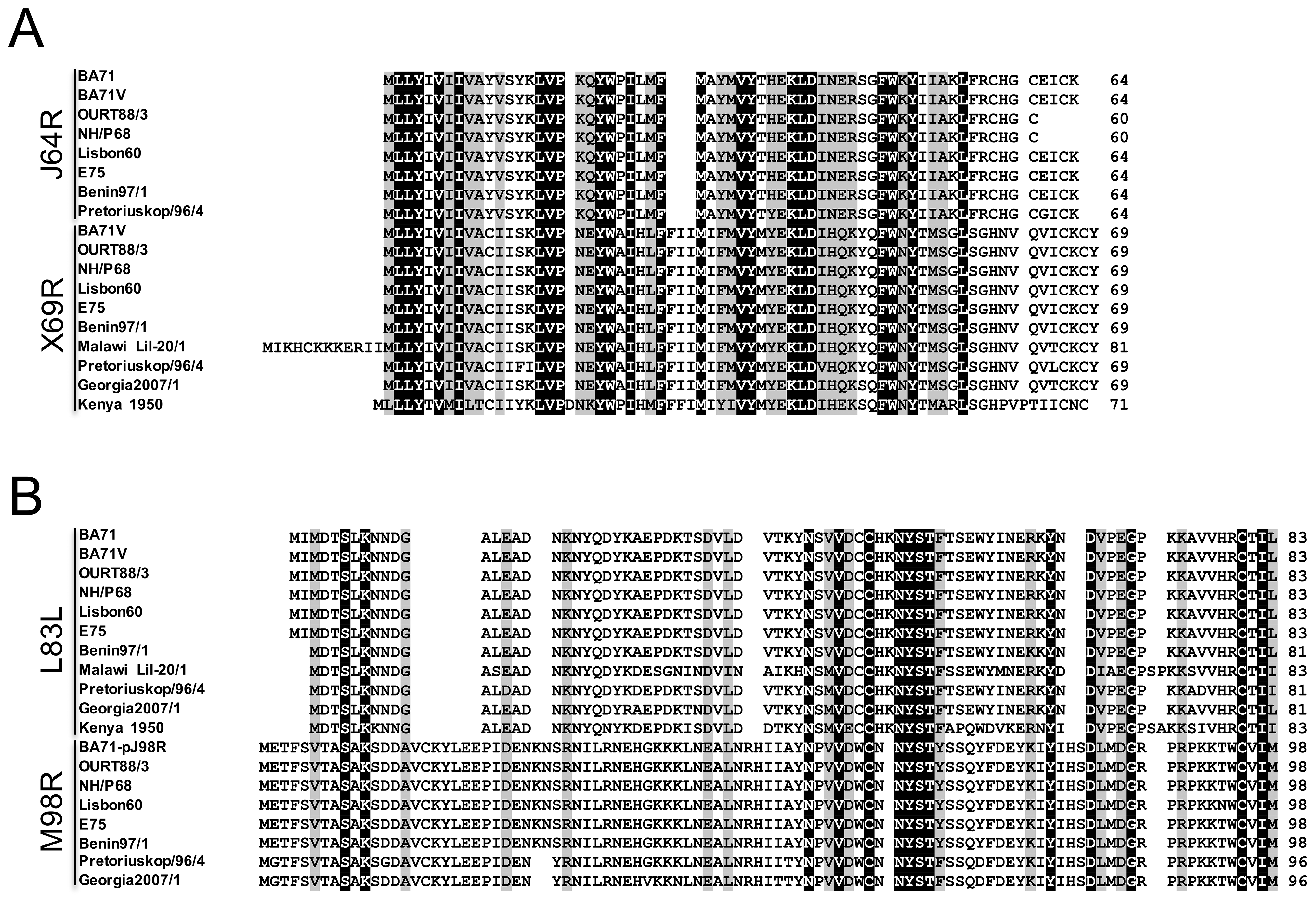

Supplement: S1 Fig — Alignment of the amino acid sequences of BA71V ORFs X69R and J64R and its orthologous in other strains. B. Sequence conservation between the amino acid sequences of ASFV ORFs L83L and M98R. Alignment of the amino acid sequences of BA71 ORFs L83L and M98R and its orthologous in other strains. Solid and shaded backgrounds indicate identical or similar amino acids, respectively. (TIF) [file pone.0142889.s001.tif]

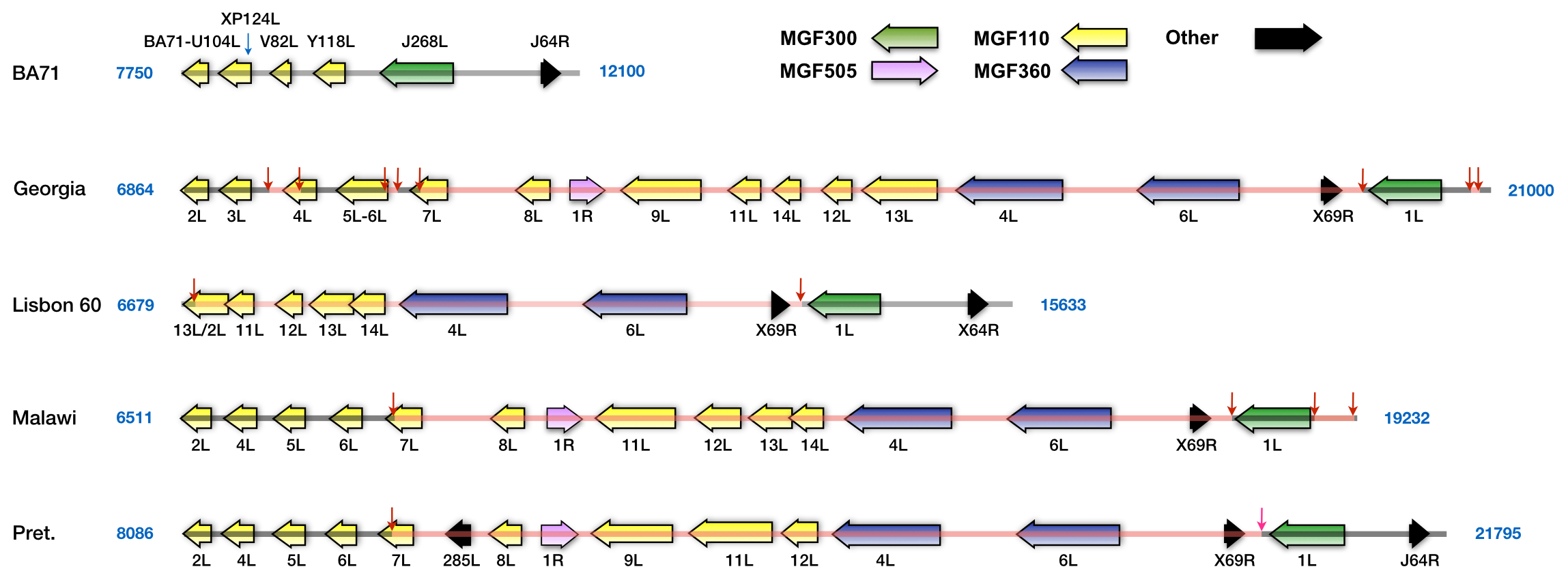

Supplement: S2 Fig — The figure shows a representation to scale of the genomes of BA71, Georgia 2007/1, Lisbon 60, Malawi Lil-20/1 and Pretoriuskop/96/4 around the position of BA71-BA71V difference 9 (the exact positions are indicated for each of the genomes). Red arrows delimit regions not present in the genome of BA71, indicated by a central red line on the genome of the virulent isolates. The different groups of ORFs are identified by colors as shown in the figure. (TIF) [file pone.0142889.s002.tif]

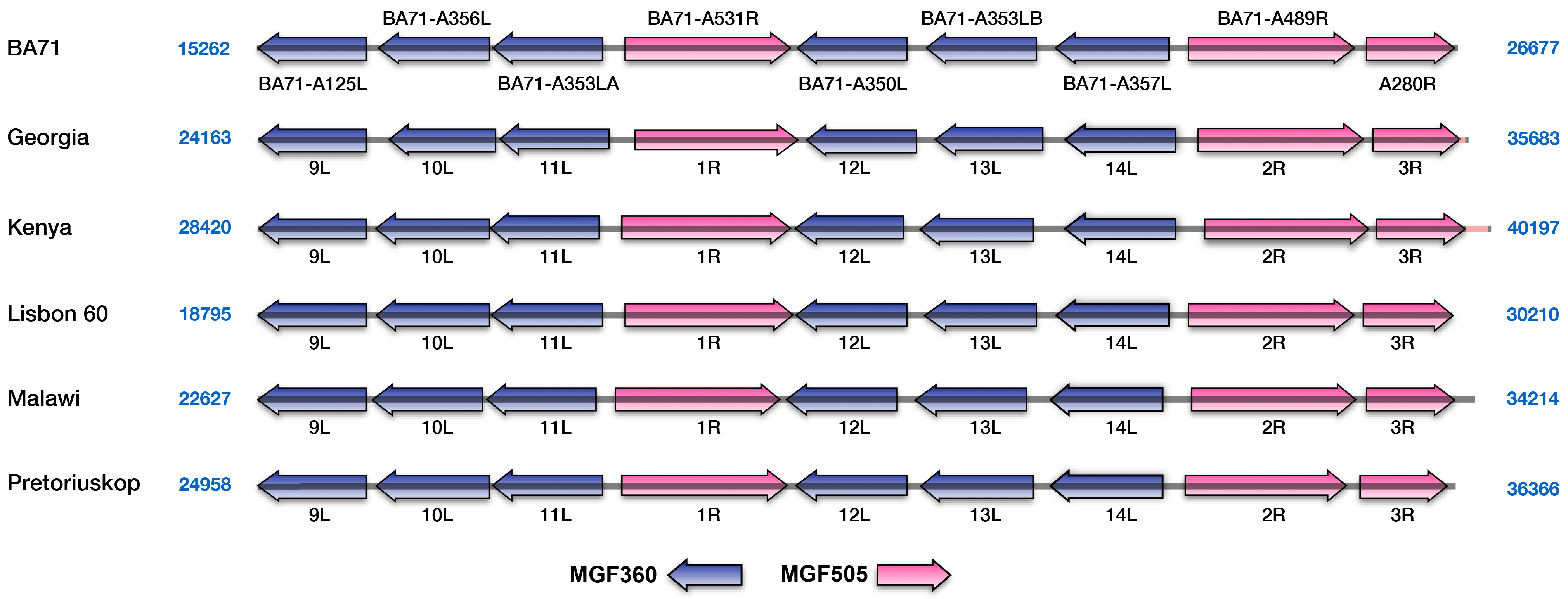

Supplement: S3 Fig — The figure shows a representation to scale of the genomes of BA71, Georgia 2007/1, Kenya 1950, Lisbon 60, Malawi Lil-20/1 and Pretoriuskop/96/4 around the position of BA71-BA71V difference 15 (the exact positions are indicated for each of the genomes). The different groups of ORFs are identified by colors as shown in the figure. (TIF) [file pone.0142889.s003.tif]

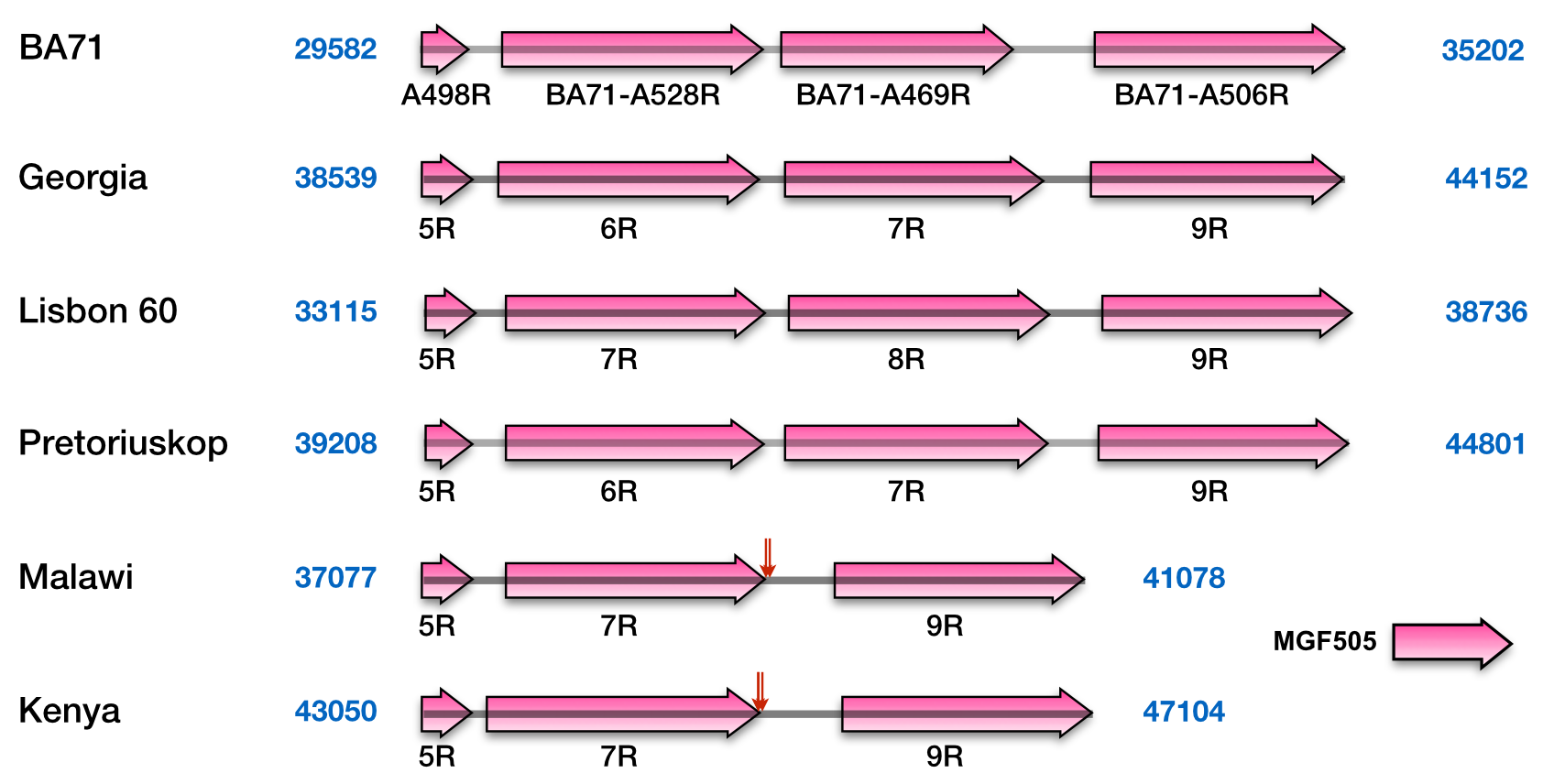

Supplement: S4 Fig — The figure shows a representation to scale of the genomes of BA71, Georgia 2007/1, Lisbon 60, Pretoriuskop/96/4, Malawi Lil-20/1 and Kenya 1950 around the position of BA71-BA71V difference 17 (the exact positions are indicated for each of the genomes). The ORFs are members of MGF 505. (TIF) [file pone.0142889.s004.tif]

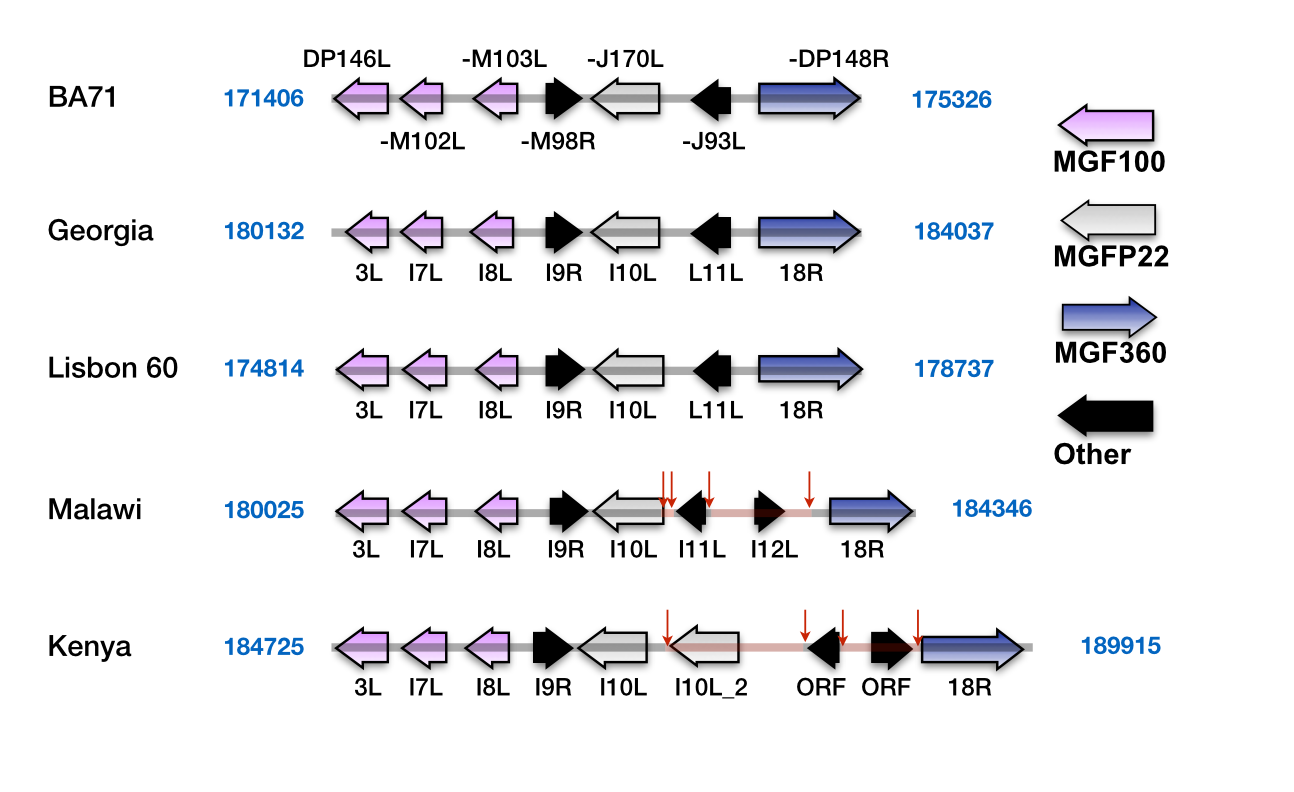

Supplement: S5 Fig — The figure shows a representation to scale of the genomes of BA71, Georgia 2007/1, Lisbon 60, Malawi Lil-20/1 and Kenya 1950 around the position of BA71-BA71V difference 49 (the exact positions are indicated for each of the genomes). Red arrows delimit regions not present in the genome of BA71, indicated by a central red line on the genome of the virulent isolates. The different groups of ORFs are identified by colors as shown in the figure. The dash at the beginning of the names of some ORFs indicates where the prefix BA71- has been removed to avoid clutter. (TIF) [file pone.0142889.s005.tif]

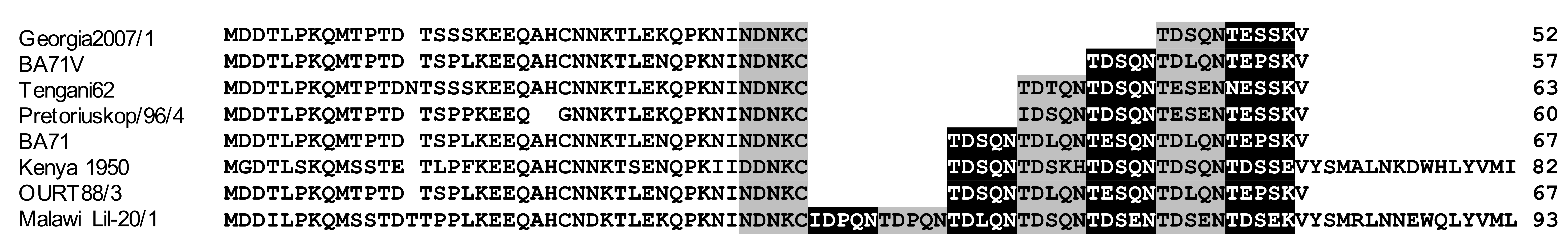

Supplement: S6 Fig — Alignment of the amino acid sequence of BA71V ORF L57L and its orthologous in other strains. The sequence of the orthologous in Lisbon 60, NH/P68, E75 and Benin97/1 is identical to that of OURT88/3. Solid and shaded backgrounds delineate the tandem repetitions. (TIF) [file pone.0142889.s006.tif]

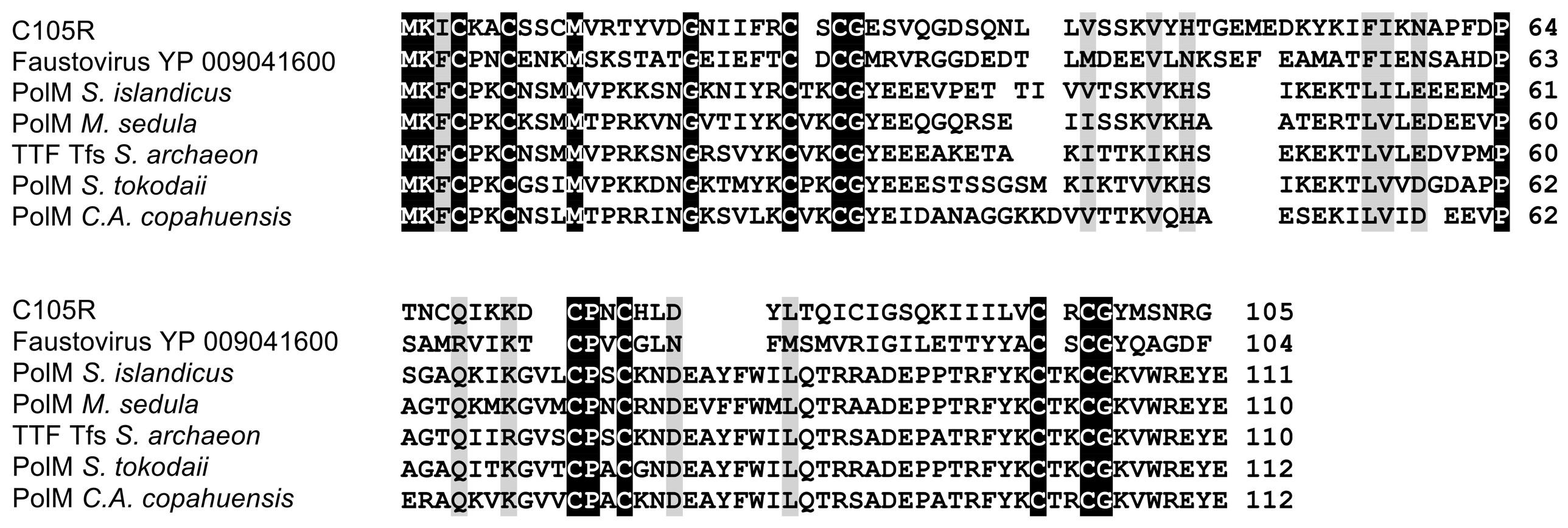

Supplement: S7 Fig — Solid and shaded backgrounds indicate identical or similar amino acids, respectively. (TIF) [file pone.0142889.s007.tif]

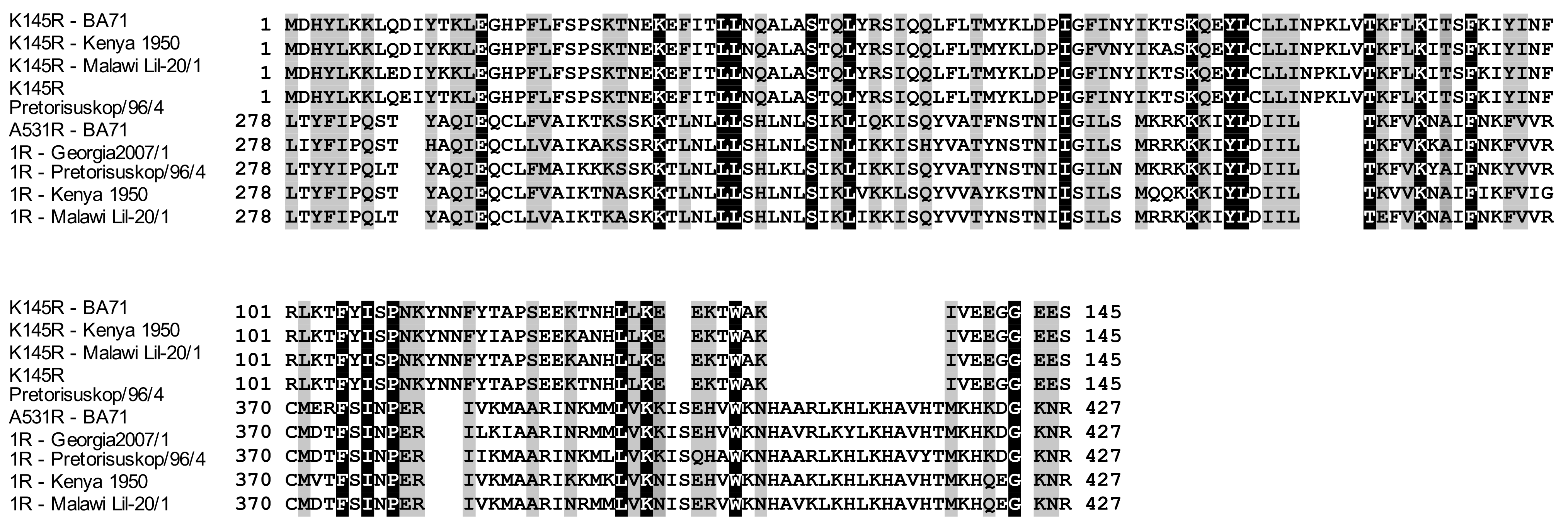

Supplement: S9 Fig — Alignment of the amino acid sequences of ASFV ORF K145R and the MGF 505 member BA71-A531R and its orthologous (1R) in other ASFV genomes. Solid and shaded backgrounds indicate identical or similar amino acids, respectively. (TIF) [file pone.0142889.s009.tif]
