# Supplementary material for: Genome Sequence of African Swine Fever Virus BA71, the Virulent Parental Strain of the Nonpathogenic and Tissue-Culture Adapted BA71V
Source: PLoS One. 2015 Nov 30;10(11):e0142889. doi: 10.1371/journal.pone.0142889 (PMC4664411; doi:10.1371/journal.pone.0142889)
Supplement: S1 Table — The table indicates the position of the changed nucleotides in the original BA71V sequence (Column U18466.1), the nature of the correction, the number of nucleotides lost or gained in each change, and the features affected by the correction. (DOCX) [file pone.0142889.s010.docx]

**Errors corrected in the sequence of BA71V**

| **U18466.1** | **Change** | **Nucleotides lost/gained** | **Affects** |
| --- | --- | --- | --- |
| **2172** | A ➔ Ø | **-1** | **TIR** |
| **2193/2194** | Ø ➔ A | **+1** | **NO ORF** |
| **2438** | C ➔ T | **-** | **KP360L (P202R)** |
| **2682** | G ➔ C | **-** | **KP360L (M283I)** |
| **2855** | G ➔ A | **-** | **KP360L (Silent)** |
| **3796** | G ➔ A | **-** | **KP362L (Silent)** |
| **5041/5042** | Ø ➔ A | **+1** | **NO ORF** |
| **12170** | T ➔ A | **-** | **J268L (C17R)** |
| **12180** | A ➔ G | **-** | **J268L (K20M)** |
| **13232** | A ➔ Ø | **-1** | **NO ORF** |
| **13543/13544** | Ø ➔ A | **+1** | **J64R** |
| **15258** | T ➔ Ø | **-1** | **NO ORF** |
| **15320/15321** | Ø ➔ T | **+1** | **J104L+J182L ➔ J328L** |
| **15685** | G ➔ Ø | **-1** |  |
| **15732/15733** | Ø ➔ C | **+1** |  |
| **67449** | C ➔ Ø | **-1** | **C122R ➔ C105R** |
| **160043-160044** | CG ➔ GC | **-** | **DP311R (R98A)** |
